# Fluoride Intake and Salivary Fluoride Retention after Using High-Fluoride Toothpaste Followed by Post-Brushing Water Rinsing and Conventional (1400–1450 ppm) Fluoride Toothpastes Used without Rinsing

**DOI:** 10.3390/ijerph192013235

**Published:** 2022-10-14

**Authors:** Justyna Opydo-Szymaczek, Tamara Pawlaczyk-Kamieńska, Maria Borysewicz-Lewicka

**Affiliations:** 1 Department of Pediatric Dentistry, Chair of Pediatric Dentistry, Poznan University of Medical Sciences, 70 Bukowska Street, 60-812 Poznan, Poland; 2 Department of Risk Group Dentistry, Chair of Pediatric Dentistry, Poznan University of Medical Sciences, 70 Bukowska Street, 60-812 Poznan, Poland

**Keywords:** salivary fluoride, toothpaste, fluoride gel, fluoride retention, fluoride intake

## Abstract

This study aimed to compare the fluoride intake and salivary fluoride levels after brushing with conventional (1400–1450 ppm) fluoride toothpaste containing different fluoride agents: amine fluoride (AmF), sodium fluoride (NaF), sodium monofluorophosphate (SMFP), potassium fluoride (KF), and a high-fluoride (5000 ppm) toothpaste containing NaF. Fourteen volunteers took part in the study. They were instructed to brush and spit without rinsing after using conventional toothpaste or brush and rinse with water after using a high-fluoride toothpaste. Fluoride concentrations were determined using ion-selective fluoride (09-37 type) in the saliva samples before and after procedures. Fluoride intake was estimated based on fluoride recovered after brushing. Additionally, the amount of fluoride present in the oral fluids and lost during the rapid clearance phase after toothbrushing was calculated based on salivary fluoride levels and saliva flow immediately after brushing. Salivary fluoride after using high-fluoride toothpaste was significantly higher than those observed after conventional products. Two hours after brushing, salivary fluoride concentrations did not differ significantly from baseline values (*p* > 0.05) unless a high-fluoride product was used. Results indicate that by refraining from rinsing after brushing with conventional toothpaste, we cannot obtain fluoride retention in saliva as with high-fluoride toothpaste. Fluoride ingestion was higher after using a high-fluoride product.

## 1. Introduction

Using oral hygiene products with fluoride content is the most common form of caries prevention [1,2]. The effectiveness of these products depends, among others, on fluoride levels in the saliva after application, which represents fluoride in the aqueous phase available for interaction with the tooth surface [3]. Salivary fluoride levels are affected by the concentration of fluoride agent applied, amount of preparation, rinsing procedure, saliva flow, and time since the exposure [4,5,6,7]. It has also been suggested that the type of fluoride agent may influence fluoride retention in saliva and bioavailability of fluoride after ingestion [8,9,10].

The level of fluoride in cosmetic OTC toothpastes and gels is restricted to 1000 to 1500 ppm fluoride, while therapeutic dentifrices, can contain up to 5000 ppm of fluoride. Prices of fluoride products may vary significantly. Toothpastes containing calcium carbonate and SMFP have usually the lowest price. Silica-based pastes tend to be more expensive, especially those high in fluorine compounds. The latter must be sold in relatively small packages, so that the total dose of fluoride in one tube does not exceed 260 mg. This is one of the factors that increase production costs. The price also depends on the additional compounds added to the product that enhance their therapeutic effectiveness. Cochrane review found a dose–response relationship between the concentration of fluoride in toothpastes and dental caries prevention [11], and prescription of high-fluoride toothpastes to prevent caries in patients at a greater risk of dental caries is recommended in many countries [11,12,13,14]. However, the cost benefit of such intervention is uncertain [13,14]. Attempts are being made to increase the effectiveness of treatments with the use of conventional toothpastes for adults and toothpastes with a reduced content of fluorides by recommendations to avoid rinsing with water after the procedure, additional use of fluoride rinse or the use of slurry remaining after the procedure in post-brushing rinsing, or adding active ingredients other than fluoride to the toothpaste [15,16,17].

On the other hand, the use of preparations with a high content of fluorides may be associated with an increase in the daily fluoride intake. Numerous studies have confirmed that each method of topical fluoride application is associated with undesirable systemic fluoride absorption [18,19,20,21,22]. Knowledge of the patient’s fluoride intake and the average salivary retention rate after using a given preparation allows us to recommend the most dose-effective topical fluoride application regimen [23,24,25,26,27,28]. Many researchers have evaluated the doses swallowed by children during toothbrushing procedures. For this purpose, fluoride or a tracer in the material that was retrieved after toothbrushing was determined [29,30,31,32,33] or the absorbed dose was estimated based on the concentration of fluoride in body fluids [18,19]. The first method has the advantage of being relatively simple and non-invasive but may lead to an overestimation of the swallowed dose since the entire portion of the formulation that cannot be recovered is considered ingested [18,29,31,34]. The second method is more complicated and time-consuming because it involves a collection of urine, blood, or ductal saliva samples [3,18,19].

There are limited data available on the ingestion of fluoride during daily hygiene routines in adults, since most studies deal with doses swallowed by children up to the age of 7 due to the risk of enamel fluorosis [29,32,33,34]. This information is useful in assessing daily fluoride intake of an adult individual. According to the U.S. Environmental Protection Agency (EPA) [35], adults in the United States consume on average 2.91 mg of fluoride from foods and beverages and 0.1 mg of fluoride from toothpaste. Lewis and Limeback [36] estimated the daily intake of fluoride from dentifrice to be 0.001 mg/kg, for adults aged over 19 years old. According to early research by Barnhart et al. who used toothpaste with a chemical tracer, adults aged 20–35 swallowed on average 2.9% of the toothpaste (0.04 g) per brushing corresponding to 0.04 mg of fluoride for toothpaste with 0.1% fluoride, with the 90th percentile at 0.12 g of toothpaste (0.12 mg of fluoride) per brushing [33].

Our previous study has shown that the concentration of fluoride in the urine of adults who switched from normal toothpaste to a paste with a fluoride content of 5000 ppm increased more than twice after two weeks of brushing. It indicates that high-fluoride toothpaste is an important source of fluoride intake [22]. It might be argued that the assessment of fluoride exposure in adults is not as important as in children, unless extremely high doses are involved that may cause skeletal fluorosis [37,38]. However, in view of the recent topical fluoride refusal and resistance, which mirrors trends seen with vaccination refusal [39], the figures constitute an important argument in the discussion. They allow patients to realize the amount of fluoride swallowed during the procedure and refer to the current recommendations regarding adequate fluoride intakes and tolerable upper intake levels (3 mg for adult females, 4 mg for males and 10 mg for adults, respectively) [37].

There are many kinds of fluoride products available on the market with different concentrations and different formulations. In everyday hygiene routines, patients aged seven years and older usually use preparations with a fluoride concentration of 1000 to 1450 ppm. In Poland, high-fluoride toothpastes are recommended only in patients from 16 year of age [40].

This study aimed to compare the salivary fluoride levels after toothbrushing with four preparations of the similar fluoride content (1400–1450 ppm), containing different fluoride agents: AmF, NaF, SMFP, and KF, as well as in one high-fluoride (5000 ppm) toothpaste containing NaF. We assumed that the type of fluoride agent may influence oral fluoride retention and that by avoiding rinsing the mouth with water after brushing with ordinary toothpaste, we could achieve fluoride retention in saliva as with high-fluoride toothpaste. We also assessed fluoride ingestion based on fluoride retrieved after the procedure, as well as the amount of fluoride eliminated from the oral cavity during the first 15 min after the procedure, which represents the dose of fluoride that would be swallowed directly after toothbrushing.

## 2. Materials and Methods

The study involved 14 volunteers from staff and students at Poznan University of Technology and Poznan University of Medical Sciences (7 women and 7 men) aged 24–40, inhabitants of the city of Poznań (Greater Poland Province), where the natural level of fluoride in the tap water has not exceeded 0.9 ppm during the last decades [41]. All subjects were in a good state of health and took no medications that might have affected their salivary flow rate.

Saliva was collected before meals or at least two hours after that. Participants were instructed not to brush with fluoride toothpaste or use any fluoride products on the study day. All samples for the analysis were collected in the dental office by the same doctor who supervised the correct performance of the study procedure.

Five fluoride preparations were used in the study: Elmex Sensitive toothpaste (Colgate-Palmolive Manufacturing Poland, Świdnica, Poland), Blend-a-med Pro Expert toothpaste (Procter & Gamble, Schwalbach, Germany), Colgate Sensitive Pro-Relief toothpaste (Colgate-Palmolive Manufacturing Poland, Świdnica, Poland), Fluor Protector gel (Ivoclar Vivadent AG, Schaan, Liechtenstein), and Duraphat 5000 (CP GABA GmbH, Hamburg, Niemcy). They contain, according to the labels, 0.14% of fluoride (in the form of the AmF Olaflur), 0.145% of fluoride (in the form of NaF), 0.145% of fluoride (in the form of SMFP), 0.145% of fluoride (in the form of KF), and 0.5% of fluoride (in the form of NaF), respectively (Table 1).

Fluoride products were purchased from supermarkets and pharmacies in the city of Poznań (3 tubes of each product). Experimental procedures involved:

1. Collection of 15 min of unstimulated saliva to assess the baseline level of fluoride in the oral fluids.
2. The 3 min application of 1 g of fluoride toothpaste or gel with the use of the toothbrush (every subject received a new toothbrush for the test). After brushing with high-fluoride product the subjects rinsed their mouth with 10 mL of distilled water and expectorated all saliva with the remaining toothpaste into the beakers. After other products, subjects did not rinse, spat out the dentifrice slurry once and put their toothbrushes heads down into plastic containers. After thorough rinsing of the toothbrushes in distilled water, the content of the containers and the contents of beakers were poured into plastic laboratory bottles, taking care that no solution remains on the walls of the vessels.
3. Collection of 15 min unstimulated saliva samples immediately after procedure, 1 h after procedure, and 2 h after procedure.

Every subject used each preparation once. Fluoride applications were performed in 2-day intervals.

Analyses of fluoride concentrations were carried out using an ion-selective fluoride electrode (09-37 type) and a RAE 111 chloride–silver reference electrode (MARAT). We did not centrifuge saliva samples, because we did not want to miss a significant portion of fluoride that resides in the sediment. Fluoride concentrations in the samples of the baseline saliva and saliva after procedures were determined using a freshly prepared calibration curve plotted by serial dilution of a concentrated stock solution of NaF (i.e., 100 mg/L). Each sample was mixed with TISAB II buffer (Merck) to maintain an appropriate ionic strength and pH.

The ionic fluoride concentrations in tubes of toothpastes and gel were assessed using the same analysis method as for the other samples, after homogenization of the product in distilled water (Table 1). The mixture was vortexed for 1 min to ensure homogeneous suspension.

Saliva flow was assessed by the gravimetric method, assuming that the specific gravity of saliva is approximately 1.0.

Fluoride concentrations in the mixture of toothpaste, saliva, and water remaining after each procedure were measured, and the dose of fluorides remaining outside the oral cavity was calculated. The amount of fluoride applied on a toothbrush was calculated based on fluoride concentrations in the product and the quantity of the product used for the procedure (1 g). Based on these data, an estimated amount of fluoride ion which a patient would eventually swallow was calculated. The amount included fluoride ingested during the procedure itself, and the part absorbed to the oral mucosa and to the teeth and dental plaque, which is slowly released into saliva and then swallowed.

Based on the salivary fluoride concentration in the first saliva sample after brushing and saliva flow, the amount of fluoride remaining in the mouth after toothbrushing and released to saliva during 15 min after procedure was calculated. It represents fluoride loosely bound to oral mucosa and teeth, swallowed soon after toothbrushing.

The data were analyzed statistically by ANOVA with post hoc pairwise comparisons using MedCalc^®^ Statistical Software version 20.013 (MedCalc Software Ltd., Ostend, Belgium; https://www.medcalc.org; 2021, accessed on 17 August 2022) with significance taken as *p* < 0.05.

## 3. Results

As seen in Table 2, there were statistically significant differences between salivary fluoride concentrations at different time points (*p* < 0.001). Fluoride concentrations immediately after toothbrushing and one hour later were significantly higher than at the baseline (*p* < 0.001). After two hours, salivary fluoride concentrations did not differ significantly from baseline values (*p* > 0.05), unless high-fluoride product was used.

Brushing with high-fluoride toothpaste resulted in the highest salivary fluoride levels immediately after procedure, significantly higher than the salivary fluoride after AMF toothpaste and SMFP toothpaste (*p* < 0.001).

At one hour and two hours after procedure, the highest salivary fluoride levels were associated with brushing with Duraphat 5000, significantly higher than after brushing with other preparations (*p* < 0.001).

Unstimulated saliva flow of the subjects during the period in the study ranged from 0.13 to 0.87 mL/min (data not listed in tables).

Fluoride intakes were significantly greater when subjects used high-fluoride toothpaste (*p* < 0.001) (Table 3).

## 4. Discussion

The results of fluoride clearance studies revealed that after a single brushing with a fluoride dentifrice, salivary fluoride decreased in two phases: an initial rapid phase which lasts from 15 to 80 min and a second slow phase lasting for several hours [7]. During slow phase, combination of continued clearance of fluoride from saliva as well as release of fluoride from various reservoirs determine current fluoride levels in oral fluids. Intra-oral fluoride reservoirs include oral mucosal surface, dental biofilm, calculus, and caries lesions [42].

According to Sotthipoka et al., the length of both phases depends on the amount of preparation applied and, to a lesser extent, on post-brushing behavior [15]. When 1 g of 1000 ppm fluoride toothpaste was used with or without rinsing, increased levels of fluoride in saliva were maintained for 60 min after brushing. Similarly, in our study, the most rapid decrease in salivary fluoride concentrations between the first and the second time interval (0–15 and 60–75 min after the procedure) and then slow gradual drop during the next 60 min of the experiment.

Rinsing with water after fluoride application reduces salivary fluoride levels [26]. This is why currently, European Academy of Pediatric Dentistry and American Academy of Pediatric Dentistry recommend that post-brushing rinsing should be reduced to a minimum in order to get the maximum beneficial effect of the fluoride exposure [43,44]. Our hypothesis was that by avoiding rinsing the mouth with water after brushing with ordinary toothpaste, we could achieve fluoride retention in saliva as with high-fluoride toothpaste. As previously revealed by Zamataro et al. [28] the salivary F bioavailability was similar when the low-fluoride (500 ppm) dentifrice was used without post-brushing rinse and the conventional fluoride dentifrice (1100 ppm) was followed by a rinse. However, our results did not confirm this assumption and only high-fluoride preparation used with rinsing provided elevation of salivary fluoride 2 h after procedure. The differences between our results and those of Zamataro et al. may be due to the much larger difference in fluoride concentrations between a conventional toothpaste and a toothpaste with a high concentration of fluoride.

Our results differ also from those obtained by Issa and Toumba [26]. In that study, two hours after brushing with any adult toothpaste (1000–1450 ppm of fluoride) without rinsing, the salivary fluoride levels were still higher than baseline levels. However, Issa and Toumba used a different study protocol, collecting the saliva for a total of 6 min during 2 h. In our study, saliva collection lasted a total of 45 min, which could lead to an accelerated elimination of fluorides from the oral cavity.

The highest fluoride levels in the first post-brushing period were observed after using high-fluoride toothpaste, then KF gel, and the lowest after SMFP dentifrice. When trying to explain the possible reasons for these results, apart from fluoride concentration, the composition of fluoride preparations should be taken into account. The semi-liquid consistency of gel may foster oral clearance of fluoride. It might have resulted in the relatively high fluoride levels in the first post-brushing saliva sample. Moreover, despite the content of calcium compound (calcium glycerophosphate), this preparation was characterized by the high availability of fluoride, which was confirmed by other studies [16,45]. Measurement of ionic fluoride concentrations revealed that they were lower than fluoride content displayed on the label (1290 vs. 1450 ppm), but higher as compared with concentrations detected in SMFP paste containing calcium carbonate (1050 ppm). In SMFP molecules, fluoride is covalently bound to phosphorus, and SMFP must be subjected to hydrolysis in the oral environment releasing fluoride ions. Thus, immediately after application of SMFP toothpaste, without additional sample preparation, a considerable proportion of fluoride cannot be detected using fluoride electrode [10,21,45,46]. Moreover, the released fluoride ion reacts with calcium carbonate as abrasive, which may inactivate part of fluoride in the toothpaste [10,47]. In the present study, we only used de-complexing techniques (TISAB) to convert all released fluoride to free ions which an ion-selective fluoride electrode can then measure. We did not use additional procedures to hydrolyze fluorine covalently bonded to phosphorus in the monofluorophosphate ion. Thus, fluoride concentration in the first saliva sample might have been underestimated. After one and two hours of SMFP hydrolysis in the oral cavity, ionic salivary fluoride concentrations were comparable to those observed after other conventional products.

In the study by Attin et al., the use of the amine fluoride dentifrice resulted in significantly higher salivary fluoride contents compared with the sodium fluoride toothpaste 90 min after toothbrushing with post-brushing rinsing [8]. It might reflect AMF properties related to special molecular structure. This type of structure, in which the fluoride ion is bound to an organic fatty acid amine fragment, is typical for tensides characterized by their surface activity, i.e., they accumulate on surfaces of all kinds [8]. In our study, the difference between salivary fluoride levels after AmF and other products was not statistically significant, although among conventional toothpastes AmF dentifrice displayed the highest salivary fluoride levels a one hour after application.

Using a similar sampling procedure, a previous study revealed that it is rather a concentration of fluoride in the preparation than the amount of preparation or method of application, which affects salivary fluoride levels after the procedure. When 1 g of toothpaste with a high concentration of fluoride (5000 ppm) was used, fluoride levels in saliva 1 h after procedure reached 2.79 ppm. When 0.6 g and 3 g of fluoride gel (12,500 ppm) were applied with the use of a toothbrush and commercial tray, fluoride levels two hours after application reached 4.04 and 4.15 ppm, respectively [5]. Similarly, Duckworth and Stewart reported a relationship between salivary fluoride levels and the applied fluoride concentration from mouthwashes [25]. The results suggested that using a given fluoride dose in a smaller volume at a higher concentration may increase the product’s efficacy. In the study by Kaczmarek and Klaniecka [27], the significantly higher increase in salivary fluoride was caused by mouthwash with a higher concentration of fluoride. At the same time, the rise of the volume and the time rinsing of the given product did not substantially influence fluoride retention.

Regarding fluoride intake, the study used two parameters to estimate the systemic fluoride exposure associated with the procedure. First parameter represented fluoride accidentally swallowed during brushing and fluoride retained in mouth. The second parameter corresponds to the portion of fluoride present in the oral fluids and loosely bound to oral mucosa which would be swallowed soon after toothbrushing. Although rinsing after brushing results in a significant decrease in fluoride ingestion and lower amount of fluoride remaining in the mouth, high-fluoride toothpaste despite rinsing was still the most significant source of fluoride.

Research from the European project “FLINT” suggest that children aged 1.5–2.5 years may swallow up to 83.9% of the amount of the paste dispensed on the toothbrush. For children aged between 2.5 and 3.5 years, this percentage was between 53% and 82% [30]. Our previous study revealed that when adults use toothpaste with a high concentration of fluoride (5000 ppm) the doses swallowed based on fluoride recovered after brushing ranged from 0.02 to 2.0 mg (mean 1.26 mg corresponding to 25% of the amount applied on the toothbrush). Similarly, in the present study, the mean percentage of fluoride not recovered after brushing amounted to 24% of the dose applied to the toothbrush [5].

Pessan et al. suggested that the actual fluoride intake from dentifrice in children may be lower than estimated in many studies. Considering fluoride intake estimated in their study based on fluoride recovered after brushing and the amount of fluoride excreted in the urine, this overestimation seems to be around 50%. This study showed that in children living in a fluoridated area, fluoride dentifrice did not cause a significant increase in the urinary fluoride output [18]. Similarly, in our previous study, we did not detect a significant increase in urinary fluoride levels in 6-year-old children after they brushed their teeth with adult fluoride toothpaste [48].

Finally, we have compared doses swallowed during the procedure and data in the literature on the total fluoride intake from diet with age-specific recommendations on Upper Tolerable Intake Levels for fluoride (UL). The UL is defined as the highest level of daily intake that is unlikely to pose risk for adverse health effects to most human individuals. Based on data on the association between fluoride intake and skeletal fluorosis, UL of 10 mg/day was established for children older than 8 years and for adults. According to the EPA [35], adults in the United States consume on average 2.9 mg of fluoride daily from foods and beverages. Thus, even assuming twice daily ingestion of 1.21 mg of fluoride during toothbrushing, the daily exposure to fluoride is still within an acceptable limit.

## 5. Conclusions

This study provides an estimation of salivary fluoride retention after the use of conventional fluoride toothpastes and gel containing different fluoride agents, and high-fluoride toothpaste. Differences between salivary fluoride levels after brushing with different products might be attributed mainly to their fluoride content, and to a lesser extent, to the different formulation of products, their consistency, and the bioavailability of fluoride agents. Two hours after brushing, fluoride levels returned to baseline, unless high-fluoride product was used. Refraining from rinsing after conventional fluoride toothpastes did not assure fluoride retention comparable to observed after using high-fluoride toothpaste. Regarding ingestion of fluoride, it was higher when subjects used high-fluoride product, despite rinsing. Nevertheless, doses swallowed during all toothbrushing procedures were negligible compared with UL.

## Figures and Tables

**Table 1:** Information about product formulation, including fluoride agents, abrasive agents, fluoride concentration declared on label [ppm], ionic fluoride measured in lab analysis (mean ± standard deviation).

Product	Fluoride Agent	Fluoride Concentration [ppm]		Abrasive Agent
		Expected	Measured	
Elmex Sensitive	AmF	1400	1380 ± 26	hydrated silica
Colgate Sensitive Pro-Relief	SMFP	1450	1050 ± 50	calcium carbonate
Blend-a-med Pro Expert	NaF	1450	1443 ± 21	hydrated silica
Fluor Protector Gel *	KF	1450	1290 ± 36	none
Duraphat 5000	NaF	5000	4990 ± 53	hydrated silica

* Contains calcium glycerophosphate.

**Table 2:** Fluoride concentrations [mg/L] in the baseline saliva, immediately after each procedure, 1 h, and 2 h after toothbrushing.

Product	Baseline (1)	0–15 min (2)	60–75 min (3)	120–135 min (4)	*p* Values
KF gel (A)	0.10 ± 0.03	8.89 ± 5.16	0.48 ± 0.27	0.12 ± 0.01	<0.001 *
Conventional NaF toothpaste (B)	0.13 ± 0.04	7.34 ± 5.64	0.61 ± 0.38	0.15 ± 0.04	<0.001 *
AmF toothpaste (C)	0.13 ± 0.04	7.15 ± 5.20	0.64 ± 0.38	0.15 ± 0.03	<0.001 *
SMFP toothpaste (D)	0.14 ± 0.03	4.85 ± 2.86	0.59 ± 0.41	0.15 ± 0.05	<0.001 *
High-fluoride NaF toothpaste (E)	0.10 ± 0.05	15.12 ± 6.33	3.63 ± 1.78	1.28 ± 0.79	<0.001 **
*p* values	0.1098	<0.001 ***	<0.001 ****	<0.001 ****	

* statistically significant differences between different time points (2) > (3) > (1),(4) (post hoc pairwise comparisons), ** statistically significant differences between different time points (2) > (3) > (4) > (1) (post hoc pairwise comparisons), *** statistically significant differences between salivary fluoride after different products (C), (D) < (E), (A) > (D) (post hoc pairwise comparisons), **** statistically significant differences between salivary fluoride after different products (E) > (A), (B), (C), (D) (post hoc pairwise comparisons).

**Table 3:** Fluoride ingestion [mg] based on the dose retrieved after brushing and fluoride retrieved from saliva after procedure.

Preparation	Fluoride Ingestion Based on the Dose Recovered after Brushing [mg] Mean ± SD	The Dose Retrieved from First Saliva Sample [mg] Mean ± SD
KF gel (A)	0.30 ± 0.10	0.063 ± 0.044
Conventional NaF toothpaste (B)	0.32 ± 0.09	0.064 ± 0.051
AmF toothpaste (C)	0.31 ± 0.08	0.054 ± 0.034
SMFP toothpaste (D)	0.30 ± 0.09	0.039 ± 0.027
High-fluoride NaF toothpaste (E)	1.21 ± 0.70	0.120 ± 0.040
*p* values	<0.001 *	<0.001 *

* Post hoc statistically significant differences (A), (B), (C), (D) < (E).

## Data Availability

The data presented in this study are available from the corresponding author on reasonable request.
